# Effects of exercise on mood changes, autonomic function and frontal alpha band lateralization in depressed university students

**DOI:** 10.3389/fpsyt.2025.1726598

**Published:** 2026-01-16

**Authors:** Zange Lin, Fengxun Lin, Meihua Su

**Affiliations:** 1 Psychological Counseling Center, Jimei University, Xiamen, Fujian, China; 2 Physical Education Institute, Jimei University, Xiamen, Fujian, China

**Keywords:** aerobic exercise, alpha band lateralization, depression, electroencephalogram, heart rate variability

## Abstract

**Objective:**

This study aims to investigate the effects of aerobic exercise on the mood, autonomic nervous system function, and frontal lobe alpha band lateralization of depressed university students, providing evidence-based references for exercise interventions in depression.

**Methods:**

34 university students with mild to moderate depression were enrolled and randomly assigned in a 1:1 ratio to either an exercise group or a control group. The exercise group undertook aerobic exercise three times weekly for 60 minutes per session over an 8-week period, while the control group maintained their usual lifestyle. Questionnaire assessments, interviews, resting electroencephalogram (EEG) recordings, and heart rate variability (HRV) measurements were employed to evaluate therapeutic differences between the two groups before and after intervention.

**Results:**

Following the 8-week intervention, the exercise group exhibited significantly reduced BDI-II scores (decrease of 11.65, P<0.05) and HAMD scores (decrease of 10.76, P<0.05), alongside decreased total frontal lobe alpha band power and markedly improved lateralization indices (P<0.05). Among time-domain indicators, the exercise group exhibited a significant reduction in HR (95%CI:-21.13,-0.52, P<0.05) RMSSD significantly increased (95%CI: 10.93, 45.31, P<0.01), while the control group exhibited a significant decrease in RMSSD (95%CI: -54.11, -7.18, P<0.05); In frequency domain metrics, the exercise group exhibited a significant reduction in LFn (95%CI: -32.58, -6.59, P<0.01), while HFn significantly increased (95%CI: 2.77, 29.81, P<0.05). The control group exhibited a significant decrease in HFn (95%CI: -25.14, -3.02, P<0.05). Correlation analysis further revealed enhanced coordination within psychological and physiological systems post-intervention, alongside emerging inter-system associations. The degree of improvement in individual depressive symptoms showed a significant negative correlation with the extent of heart rate reduction (r_s_= -0.684, P = 0.002), suggesting a potential synchronous mechanism underpinning both improvements.

**Conclusion:**

Improvements in autonomic nervous function and frontal lobe EEG metrics indicate that long-term, regular aerobic exercise can alleviate depressive mood in university students, enhance frontal lobe brain function, and balance sympathetic and parasympathetic nervous system activity. This holds promise as an effective method for alleviating depression. This study provides preliminary evidence supporting structured exercise programs as a campus-based supplementary intervention for depression among university students.

## 1 Introduction

Major depressive disorder (MDD) is a prevalent brain dysfunction that can lead to sustained low mood, impaired concentration, and diminished executive function during depressive episodes. In severe cases, it is accompanied by physical impairments and even suicidal behavior. According to the World Health Organization, the lifetime prevalence of MDD is 17%, affecting approximately 350 million people globally. Projections indicate that by 2030, the medical burden and impact on global health caused by depression will rank first (1). In recent years, the proportion of university students experiencing mental health issues has risen steadily, stemming from factors such as leaving home for study, navigating social interactions, and adapting to academic pressures (2). University students have emerged as one of the groups with a high incidence of depression. Research indicates that the detection rate of depression among university students reaches as high as 24.71%, with suicide rates attributable to depression increasing annually. MDD has now become one of the significant mental health challenges severely affecting university students’ psychological wellbeing (3). Electroencephalography (EEG), as a non-invasive, safe, and side-effect-free method for studying cortical neurophysiology, employs frontal alpha asymmetry as a quantitative indicator of functional disharmony between brain hemispheres. Most studies identify frontal alpha asymmetry as a key ‘biomarker’ potentially reflecting human emotions, applicable for early depression diagnosis (4). Furthermore, individuals with MDD frequently exhibit autonomic nervous system dysfunction, manifesting as cortical dysregulation of autonomic functions and resulting in sympathetic-parasympathetic imbalance. Heart rate variability (HRV) reflects the autonomic nervous system’s regulation of cardiac sinus node activity by sympathetic and parasympathetic nerves, serving as an indicator of autonomic functional tension and equilibrium (5). Studies demonstrate significantly reduced HRV in MDD patients, with more pronounced declines observed in those experiencing anxiety (6). The more severe the symptoms in MDD patients, the more pronounced the reduction in their HRV levels (7). For patients currently in the slow phase of a depressive episode, a significant and sustained decline in HRV may serve as a precursor to relapse (8). Furthermore, HRV can function as a biological marker for predicting MDD onset and for evaluating the efficacy of treatments aimed at alleviating depressive symptoms.

In summary, physiological dysregulation in individuals with depression manifests as abnormal frontal lobe alpha asymmetry and reduced HRV. Neuroscience research indicates that HRV and frontal lobe alpha asymmetry are not mutually independent phenomena. The prefrontal cortex, serving as a higher-order center for emotional and cognitive regulation, exerts top-down modulation over cardiac autonomic activity via the autonomic nervous system (9). The functionality of this brain-heart regulatory pathway relies upon a healthy neurochemical environment, encompassing neurotrophic factors that promote neuronal health and neurotransmitters such as serotonin that foster emotional stability (10). Consequently, prefrontal dysfunction in depression may lead to diminished regulation of autonomic nervous system function, precipitating a decline in HRV.

In recent years, exercise therapy has emerged as a crucial non-pharmacological intervention in the adjunctive treatment of depression. Physical activity significantly ameliorates depressive states (11) and garners considerable attention within the field due to its high compliance, low treatment costs, minimal adverse effects, and efficient, stable antidepressant efficacy. Research indicates that high-intensity interval training demonstrates superior antidepressant effects compared to moderate-intensity exercise, while also exhibiting significant efficacy in sustaining these benefits (12). Regular physical activity enhances cardiac autonomic function, manifested through increased HRV (13). Concurrently, evidence suggests aerobic exercise modulates EEG activity patterns in the prefrontal cortex, improving asymmetry in frontal alpha waves (14). Exercise may represent a key pathway for improving cortical and autonomic nervous system function, though its precise mechanisms in treating depression remain unclear. Current research predominantly examines exercise’s isolated effects on either the autonomic nervous system or EEG activity, without integrating these dual influences. Although exercise therapy shows potential in treating depression, existing research is limited by its homogeneity in exercise type and organizational format, potentially constraining its efficacy. One meta-analysis indicates that mixed group exercise interventions yield superior outcomes for depressed adolescents compared to isolated individual aerobic exercise (15). However, such programs often face challenges such as fixed intensity and lack of progression, rendering them ill-suited to patients’ fluctuating physiological and psychological states. Based on this, the present study designed an 8-week group aerobic exercise program for 34 university students with mild-to-moderate depression. The program comprised exercises such as jumping jacks and burpees, with progressively increasing intensity. The study examined changes in participants’ mood, autonomic nervous system function, and frontal lobe alpha band lateralization before and after the intervention. It aimed to elucidate the brain’s biological mechanisms underlying exercise therapy’s efficacy in alleviating depression among university students, thereby providing more integrated and adaptable practical evidence for non-pharmacological interventions in depression management.

## 2 Materials and methods

### 2.1 Inclusion and exclusion of study participants

This study adhered to the principles of a randomized controlled trial design. The recruitment center was the Psychological Counselling Centre of Jimei University, selecting university students meeting diagnostic criteria for unipolar depression as research subjects. Participants were recruited through online and offline promotional channels and randomly assigned using computerized random number generation to either the control group (CON) or the exercise group (EXD). This study was approved by the Jimei University Science and Technology Ethics Committee, with ethics approval number JMU202501001. All participants were fully informed of the project’s content and associated risks and provided written informed consent.

Inclusion criteria: According to the Diagnostic and Statistical Manual of Mental Disorders, Fifth Edition (DSM-5) criteria for depression (16), combined with the Beck Depression Inventory. Depression Self-Rating Scale (BDI-II) (17) (depression level ≥15 points) and the Hamilton Depression Rating Scale (HAMD) (18) (total score ≥8 points as assessed by others); subjects were aged 18–25 years, right-handed, and in good physical health.

Exclusion criteria: Exclude individuals with organic brain disorders, substance dependence, severe physical illnesses, pregnant or lactating women, and those with regular high-intensity exercise habits, while also ensuring no prior use of psychotropic medications such as barbiturates or benzodiazepines.

### 2.2 Experimental intervention process

#### 2.2.1 Randomization and study grouping

**Figure 1:**
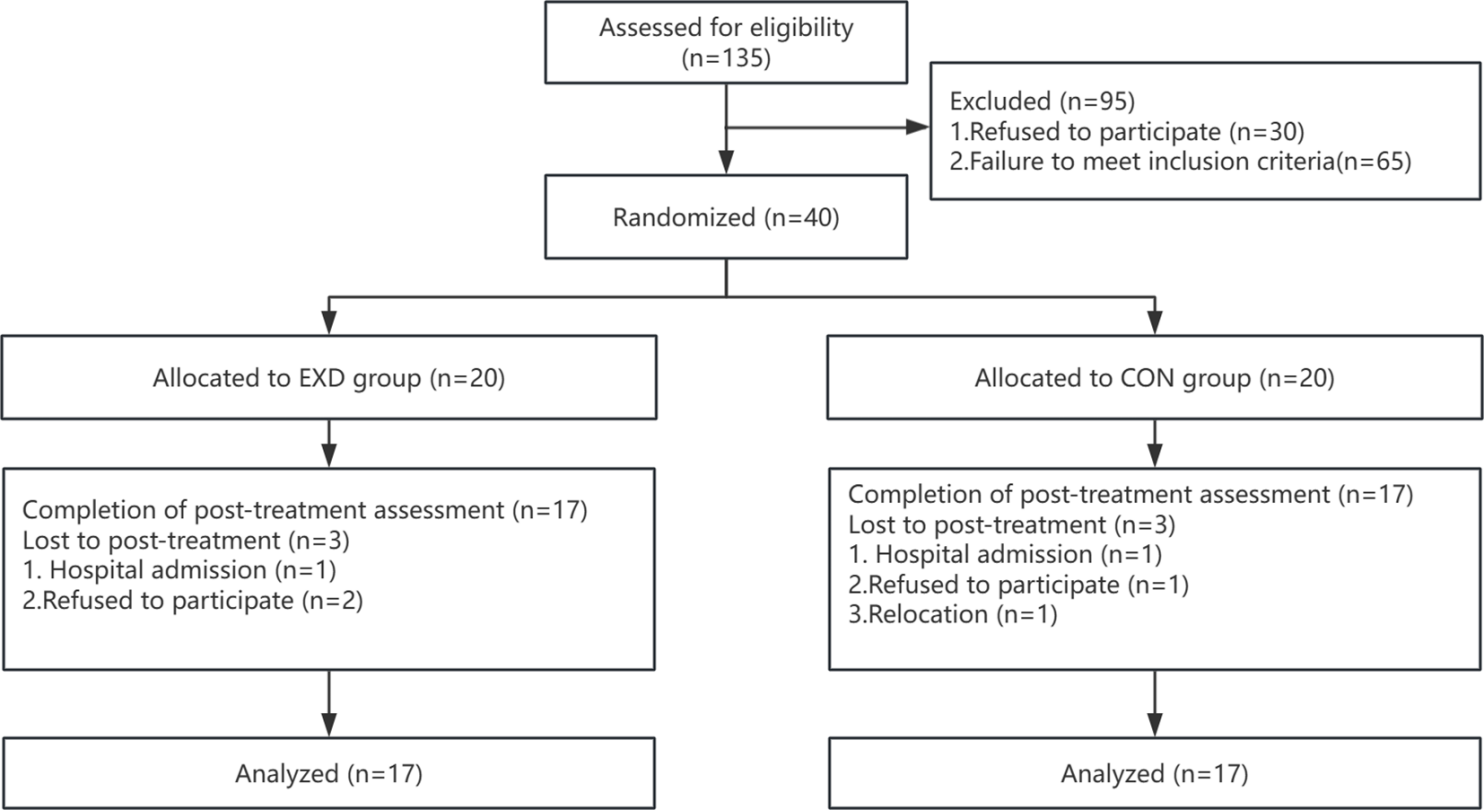
Experimental participation process.

Participants were randomly assigned in a 1:1 ratio to the CON group and EXD group using a random number generator (19). This study employed a single-blind design for the assessors: the researchers responsible for collecting and analyzing all physiological indicators and questionnaire scores were completely unaware of the participants’ group assignments, whereas the participants and exercise intervention practitioners were informed of their groupings. The experimental participation process for subjects is illustrated in **Figure 1**.

Control group (CON group): No intervention; continued with existing treatment regimen.

Exercise group (EXD group): Received aerobic exercise in addition to existing treatment, comprising 8 weeks of training, 3 days per week, 60 minutes per session. 

#### 2.2.2 Exercise intervention protocol

Participants in the EXD group underwent an 8-week program comprising three 60-minute in-person training sessions per week. Training primarily consisted of aerobic exercise, with intensity monitored in real-time via heart rate monitoring during sessions. A heart rate armband (Miopod integrated heart rate armband, Shuoda Health Technology Co., Ltd.) was used to measure participants’ maximum heart rate (HRmax). Maximal heart rate was calculated using Gellish et al.’s formula: ‘HRmax = 207 - 0.7 × age (20). Target intensity levels were defined according to the American College of Sports Medicine (ACSM) guidelines: low intensity (57-63% HRmax), moderate intensity (64-76% HRmax), and high intensity (77-95% HRmax) (21). Each training session commenced with a 10-minute warm-up at low-to-moderate intensity followed by low-intensity cool-down stretches. Heart rate protocols were progressively escalated over the 8-week program as detailed below:

Week 1 served as an adaptation phase, comprising three instructional sessions to enable subjects to master the fundamental movements. Each exercise employed progressive decomposition training, beginning with isolated component movements before progressing to full-range execution. Each movement was performed for 40 seconds, followed by 40 seconds of rest, repeated for 2 sets, maintaining heart rate between 120–140 bpm. Formal exercise intervention commenced thereafter for the subsequent eight weeks. Weeks 2–5 training schedule: Each exercise performed for 40 seconds, followed by 30 seconds rest, repeated for 3 sets. Heart rate maintained between 120–160 bpm. Weeks 6–8 training schedule: Each exercise performed for 40 seconds, followed by 20 seconds rest, repeated for 4 sets. Heart rate maintained between 170–190 bpm.

Throughout the program, each participant was permitted three absences; exceeding this limit resulted in withdrawal. Training days were scheduled for Tuesdays, Thursdays, and Saturdays, with no exercise intervention on other days. The detailed training protocol is outlined in **Table 1**.

**Table 1:** Training program for weeks 1-9.

Training program	Preparatory part	Fundamental part	Concluding part
Week 1 (adaptation period)	1.5min jogging after gathering the whole team. 2. Warm up after jogging, including head movement, shoulder loop, chest expansion, hip movement, left and right lunge leg press, knee movement, wrist and ankle movement, four eight beats for each movement.	Exercises: open and close jumps, high kicks, burpees, lunge jumps, push-ups, squats, planks, mountain running 40 seconds for each movement, 40 seconds rest, repeat for 2 sets.	Subjects were organized to perform static stretches Includes: neck muscle stretch, shoulder stretch, biceps stretch, triceps stretch, gluteus maximus stretch, quadriceps stretch, biceps stretch, gastrocnemius stretch, 30 seconds for each movement, both include left and right sections
Weeks 2-5		Exercises: open and close jumps, high kicks, burpees, lunge jumps, push-ups, squats, planks, mountain running 40 seconds for each movement, 30 seconds rest, repeat for 3 sets.	
Weeks 6-9		Exercises: open and close jumps, high kicks, burpees, lunge jumps, push-ups, squats, planks, mountain running 40 seconds for each movement, 20 seconds rest, repeat for 4 sets.	

### 2.3 Intervention outcome indicators

#### 2.3.1 Basic information survey

Face-to-face interviews were conducted by investigators who had undergone standardized training and passed assessment. The basic information and demographic questionnaire utilized a self-designed instrument, primarily covering: basic details (measuring height and weight), lifestyle and behavioral patterns (smoking history, drinking history, etc.), past medical history, and basic household circumstances.

Physical activity levels were assessed using the International Physical Activity Questionnaire (IPAQ) Short Form, collecting data on intensity, duration, and frequency of physical activity over the preceding seven days. Total physical activity was calculated according to the IPAQ Calculation Manual using the formula: Physical Activity Volume (MET-min/week) = MET value × duration (minutes)/day × number of active days (22).

#### 2.3.2 Assessment of mood state

The BDI-II was revised by Beck et al. based on the first edition and serves to assess depressive symptoms and their severity over the preceding fortnight in individuals with mental disorders, reflecting patients’ internal cognitive and emotional experiences (17). The HAMD-17 objectively evaluates somatic and behavioral symptoms in patients with depression. This scale demonstrates good internal consistency, with Cronbach’s alpha values ranging from 0.70 to 0.84 (23). This study employed the Beck Depression Inventory-II (BDI-II) and Hamilton Depression Rating Scale (HAMD-17) pre- and post-intervention to evaluate mood state changes following exercise intervention. Assessments were conducted via face-to-face interviews by specialist clinicians who had undergone standardized training and passed certification examinations, thereby ensuring the precision of mood state evaluations.

#### 2.3.3 Electroencephalogram signal acquisition

**Figure 2:**
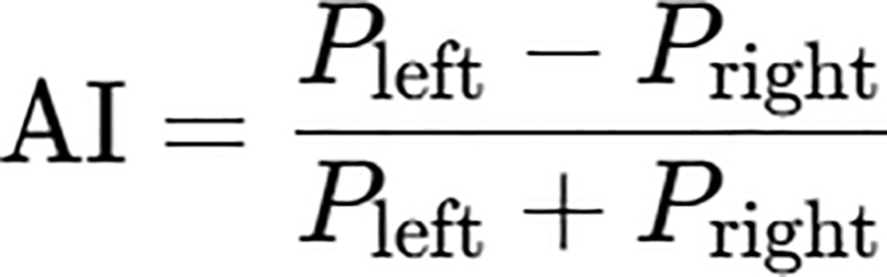
Formula for Alpha wave asymmetry in the brain.

This study employed an EEG recording device manufactured by Fuzhou Hongrui Health Technology Co., Ltd., model: NM-8C. EEG signals were acquired via saline-filled electrodes placed on the scalp. The A1 and A2 leads were connected to the mastoid processes of the left and right ears respectively, serving as the reference and ground electrodes, to collect an 8-channel EEG signal. Electrode placement followed the International EEG Society’s 10/20 system, positioned at Fp1, Fp2, F3, F4, P3, P4, O1, and O2. Parameters were set as follows: sampling frequency 500Hz, high-pass filter 0.5Hz, low-pass filter 40Hz, and power-frequency notch filter at 50Hz. Three days prior to testing, subjects were instructed to abstain from caffeinated beverages, ensure adequate sleep, and refrain from strenuous exercise the day before testing. Electrodes were soaked in physiological saline for 30 minutes prior to testing. Upon arrival at the laboratory, subjects should rest quietly for 15 minutes. Connect all EEG leads, switch on the power supply, launch the EEG acquisition software, fit the EEG cap, and input the subject’s identification number. Instruct subjects to relax while remaining alert, avoiding clenching teeth or blinking, and maintaining steady breathing. EEG signal acquisition utilizes an 8-channel configuration divided into four brain regions: the frontal lobe (left and right frontal areas), parietal lobe, and occipital lobe (left and right occipital areas). The corresponding electrodes are Fp1, Fp2, F3, F4, P3, P4, O1, and O2. The acquired EEG signals undergo initial filtering: a 0.5Hz high-pass filter removes DC drift and low-frequency motion artefacts; a 40Hz low-pass filter eliminates high-frequency noise; and a 50Hz notch filter suppresses mains frequency electrical noise. This enhances the reliability of subsequent feature extraction and classification. Simultaneously, Independent Component Analysis (ICA) is employed to eliminate EOC artefacts, isolating electrooculographic components (e.g., blinking, eye movements). Subsequently, EEG analysis software pre-processes the signals and applies Fourier transforms to calculate the average power spectral values for six rhythms (Delta, Theta, Alpha, Low-Beta, High-Beta, Gamma) across six rhythms. Power within the 8–13 Hz band is defined as Alpha power. Alpha waves (8–13 Hz) constitute the most prevalent rhythm in the occipital region during adult quiet, awake, and relaxed states, typically associated with functions such as emotional regulation. Frontal alpha asymmetry was calculated using the symmetrical electrodes F3 (left frontal lobe) and F4 (right frontal lobe) as representative electrodes. Left frontal alpha total power (Pleft): the average of the power spectrum in the alpha band (8–13 Hz) for electrode F3; Right frontal alpha total power Pright: average power spectrum value in the alpha band (8–13 Hz) from electrode F4; Total frontal alpha power is Pleft + Pright, representing overall frontal alpha activity levels; This study calculated the Asymmetry Index (AI) (24) for cerebral alpha waves using the formula in **Figure 2**.

#### 2.3.4 Heart rate variability acquisition

HRV serves as a key metric for assessing autonomic nervous system activity, encapsulating extensive regulatory information on neurohumoral influences on the cardiovascular system. It quantitatively evaluates the tensions of sympathetic and parasympathetic nerves and their balance. Additionally, HRV can assess parasympathetic nerve tension in individuals with depression. Schaffarczyk et al (25) employed the Polar H10 and a 12-channel electrocardiogram (CardioPart 500) to assess heart rate variability in 14 adults. The findings demonstrated a perfect correlation (R²= 1.00) between the R-R interval and resting heart rate across both devices during the same acquisition period, underscoring the Polar H10’s strong usability in HRV data collection and supporting its theoretical application. In this study, the Polar H10 device from the Netherlands was utilized to measure HRV in participants. To ensure accuracy, subjects abstained from tea and coffee consumption prior to testing. Both pre- and post-tests were conducted in the same room, maintained at a constant temperature of 25 °C. During the test, subjects remained quiet, relaxed, and motionless in a supine position. Test precautions were explained beforehand. A heart rate chest strap was placed at the 3rd-4th intercostal space along the left mid-clavicular line. Subjects then closed their eyes and lay flat for approximately 5 minutes. After cleaning the chest skin, the Polar H10 connection was adjusted. Electrocardiogram (ECG) data of the R-R interval were collected at a 1000 Hz sampling rate, with real-time monitoring of the R-R interval waveform. Signal quality was assessed using Polar Flow software. If baseline drift or motion artifacts, such as those caused by muscle tremors, were detected, recording was paused and the electrode position adjusted. ECG data were processed using the Pan-Tompkins Algorithm and corrected with a semi-automatic method, which involved setting a threshold for checking, cleaning, deleting, and manually inspecting and marking the cardiac R waves (26). The HRV data were preprocessed using Kubios software with default settings. The detrending method for R-R intervals was set to Smoothn priors, and the smoothing parameter Lambda was set to 500. After pre - processing, data analysis was carried out. Subsequently, the following HRV parameters were calculated: the overall standard deviation of normal sinus R-R intervals (SDNN), the root mean square of adjacent R-R interval differences (RMSSD), high frequency power (HF), low frequency power (LF), and the ratio of low frequency to high frequency power (LF/HF). The HF and LF values were normalized units (n.u.), calculated as HF (n.u.) = HF[ms²]/(total power[ms²] - very low frequency (VLF)[ms²]) and LF (n.u.) = LF[ms²]/(total power[ms²] - VLF[ms²]).

### 2.4 Statistical analysis

Data entry, cleaning, and statistical analysis were conducted using SPSS 22.0 software. Normality tests were first performed on the data. Where data met the criteria for normal distribution, between-group baseline comparisons employed an independent samples t-test, while within-group pre- and post-intervention comparisons utilized a paired samples t-test. Results were expressed as mean ± standard deviation, reporting the mean difference and its 95% confidence interval for within-group pre- and post-intervention changes. Where data did not meet normality assumptions, results were presented as M (P25, P75). Between-group comparisons employed the independent samples rank sum test, while within-group pre- and post-intervention comparisons used the paired samples rank sum test. To investigate associations between psychological scale scores, electroencephalographic (EEG) indicators, and heart rate variability (HRV) measures, Spearman’s rank correlation analysis was employed due to the small sample size and potential non-normality of certain variables. The correlation coefficient (r_s_) was calculated to assess the strength and direction of associations. The significance level was set at α=0.05, with P<0.05 indicating statistically significant differences.

## 3 Results

### 3.1 Basic information of the enrolled participants

After the initial screening, a total of 40 participants were included in the study. Three participants withdrew from both the control group and the experimental group. Finally, 34 participants completed all the experiments and tests. Among them, the number of participants in the exercise group (EXD group) was 17, and that in the control group (CON group) was also 17, as shown in **Figure 2**. According to **Table 2**, there were no significant statistical differences between the two groups of participants in terms of age, gender, height, weight, BMI, duration of depressive episodes, number of depressive episodes, smoking, alcohol consumption, and whether they were only - children (p > 0.05).

**Table 2:** Basic and demographic information of the included participants.

Baseline indicators	Categorization	EXD group (n=17)	CON group (n=17)	Statistical value	P-value
Age[year]	–	19.06 ± 1.09	18.59 ± 0.80	t = -1.44	0.160
Gender [n (%)]	Male	7 (41.17%)	10 (58.82%)		
	Female	10 (58.82%)	7 (41.17%)		
Height [cm]	–	168.32 ± 7.77	168.50 ± 7.39	t = 0.07	0.946
Weight [kg]	–	73.38 ± 8.40	71.05 ± 10.01	t = -0.74	0.467
BMI[kg/cm2]	–	25.86 ± 2.14	25.86 ± 2.14	t = -1.22	0.231
Duration of depressive episodes (months)		3.00 (2.00, 5.00)	2.00 (2.00, 4.00)	Z= -0.37	0.709
Number of depressive episodes (times)		2.00 (1.00, 2.00)	1.00 (1.00, 2.00)	Z= -0.07	0.940
Smoking [n (%)]	No	15 (88.23%)	16 (94.11%)	X2= -0.60	0.551
	Yes	2 (11.76%)	1 (5.88%)		
Drinking [n (%)]	Yes	4 (23.52%)	4 (23.52%)	X2 = 0.00	1.000
	No	13 (76.47%)	13 (76.47%)		
an only child	Yes	10 (58.82%)	11 (64.70%)	X2=-0.35	0.728
	No	7 (41.17%)	6 (35.29%)		
Physical activity level (MET-min*week-1)	–	517 (318,759)	687 (240,986)	Z = -0.29	0.770

### 3.2 Comparison of the changes in the state of mind of the two groups of subjects before and after the intervention

**Table 3** demonstrates comparable baseline scores on the BDI and HAMD scales between the two subject groups (P > 0.05). Following an 8-week intervention, a significant reduction in BDI and HAMD scores was observed in the EXD group compared to pre-intervention levels (P < 0.05), while no significant change was noted in the control group. Semi-structured interviews revealed that within the EXD group, 12 individuals (70.58%) reported improved mood, 4 individuals (23.52%) experienced decreased negative emotions, and 1 individual (5.88%) reported no change. In contrast, within the CON group, 3 individuals (17.64%) reported enhanced mood, 1 individual (5.88%) noted reduced negative emotions, and 13 individuals (76.47%) reported no change, which indicated that aerobic exercise compliance was indicating high adherence to aerobic exercise and good therapeutic effect.

**Table 3:** Comparison of the state of mind of the two groups of subjects before and after the intervention.

Indicators	Groups	Pre-intervention	Post-intervention	Difference	t-value	P-value
BDI Score	CON Group	20.35 ± 3.26	17.94 ± 6.25	-2.41 ± 6.17	1.61	0.126
	EXD Group	21.71 ± 5.13	10.06 ± 5.18	-11.65 ± 5.92	8.12	0.001
t-value		-0.92				
P-value		0.366				
HAMD Score	CON Group	16.24 ± 1.75	13.88 ± 4.92	-2.35 ± 4.94	1.97	0.067
	EXD Group	17.82 ± 3.13	7.06 ± 3.98	17.82 ± 3.13	12.84	0.001
t-value		-1.83				
P-value		0.077				

### 3.3 Comparison of frontal alpha band lateralization before and after intervention in the two groups of subjects

As shown in **Tables 4**, **5**, before the intervention, there were no statistically significant differences in the total power values of the left and right frontal lobe α frequency bands and the lateralization of the frontal lobe α frequency bands between the two groups of subjects, indicating consistency at the baseline level. Following the 8-week intervention, the exercise group exhibited a decrease in the total power of the frontal lobe α frequency band and a significant improvement in its lateralization (P < 0.05). In contrast, the control group showed no significant changes in these metrics post-intervention.

**Table 4:** Comparison of frontal alpha band total power values before and after intervention in the two groups of subjects.

Groups	Fp1+F3				Fp2+F4			
	Pre-intervention	Post-intervention	t-value	P-value	Pre-intervention	Post-intervention	t-value	P-value
EXD Group	186.45 ± 114.67	129.82 ± 52.96	2.22	0.041*	190.09 ± 117.01	133.20 ± 67.32	2.20	0.043*
CON Group	152.21 ± 61.61	173.25 ± 81.64	-1.10	0.287	150.80 ± 62.14	193.93 ± 101.68	-1.88	0.094
t-value	1.04				0.35			
P-value	0.358				0.293			

Fp1+F3 are left frontal total power values, Fp2+F4 are right frontal total power values *P<0.05.

**Table 5:** Comparison of frontal alpha band lateralization before and after intervention in the two groups of subjects.

Groups	Fp1-Fp2				F3-F4			
	Pre-intervention	Post-intervention	t-value	P-value	Pre-intervention	Post-intervention	t-value	P-value
EXD Group	0.20 ± 0.35	0.03 ± 0.07	2.14	0.048*	0.16 ± 0.26	0.02 ± 0.06	2.12	0.05*
CON Group	0.02 ± 0.10	0.05 ± 0.07	-1.05	0.308	0.01 ± 0.15	0.05 ± 0.10	-1.07	0.299
t-value	1.66				1.17			
P-value	0.106				0.249			

Fp1-Fp2, F3-F4 are α-band lateralized, *P<0.05.

### 3.4 Comparison of autonomic function before and after intervention in the two groups of subjects

As shown in **Table 6**, no statistically significant differences were observed between the two groups in either the time-domain parameters (HR, RMSSD, SDNN) or frequency-domain parameters (LFn, HFn, LF/HF) prior to intervention, indicating consistency in baseline levels between participants. Following the 8-week intervention, among the time-domain parameters, the exercise group exhibited a significant decrease in HR compared to pre-intervention levels (95% CI: -21.13, -0.52, p < 0.05), while RMSSD significantly increased (95% CI: 10.93, 45.31, p < 0.01). In the control group, RMSSD significantly decreased (95% CI: -54.11, -7.18, p < 0.05). In the frequency domain metrics, the exercise group exhibited a significant reduction in LFn compared to pre-intervention levels (95% CI: -32.58, -6.59, p<0.01), while HFn significantly increased (95% CI: 2.77, 29.81, p<0.05). Conversely, the control group exhibited a significant decrease in HFn (95% CI: -25.14, -3.02, p<0.05). The LF/HF ratio in both groups showed no statistically significant change before and after the intervention (p>0.05).

**Table 6:** Comparison of autonomic function before and after intervention between the two groups of subjects.

Indicators	Groups	Pre-intervention	Post-intervention	Change (mean ± SD)	95%CI	t-value	P-value
time domain index							
HR (bmp)	CON	81.82 ± 17.09	80.47 ± 11.91	-1.35 ± 26.45	(-14.95,12.25)	-0.21	0.836
	EXD	88.12 ± 15.17	77.29 ± 12.09	-10.82 ± 20.04	(-21.13,-0.52)	-2.23	0.041*
RMSSD (ms2)	CON	55.35 ± 43.54	24.71 ± 15.23	-30.65 ± 45.64	(-54.11,-7.18)	-2.77	0.014*
	EXD	35.44 ± 34.17	64.13 ± 45.12	28.12 ± 33.43	(10.93,45.31)	3.47	0.005#
SDNN (ms2)	CON	24.47 ± 17.38	24.18 ± 15.84	-0.30 ± 21.65	(-11.43,10.83)	-0.06	0.956
	EXD	24.47 ± 17.38	37.50 ± 25.59	13.03 ± 32.30	(-3.58,29.64)	1.66	0.116
frequency domain index							
LFn	CON	51.72 ± 20.96	62.63 ± 19.88	10.90 ± 29.82	(-4.43,26.24)	1.51	0.151
	EXD	67.99 ± 15.25	48.41 ± 19.42	-19.59 ± 25.28	(-32.58,-6.59)	-3.20	0.006#
HFn	CON	51.41 ± 19.45	37.33 ± 19.88	-14.08 ± 21.51	(-25.14,-3.02)	-2.70	0.016*
	EXD	31.89 ± 15.22	48.18 ± 20.99	16.29 ± 26.29	(2.77,29.81)	2.55	0.021*
LF/HF	CON	1.32 ± 1.19	3.03 ± 3.33	1.71 ± 3.36	(-0.02,3.44)	2.09	0.053
	EXD	3.23 ± 2.98	1.47 ± 1.07	-1.76 ± 3.43	(-3.52,0.01)	-2.11	0.051

*P<0.05, #P<0.01

### 3.5 Comparison of Spearman correlation analysis results between variables before and after intervention

As shown in **Table 7**, the correlation between various indicators underwent a systematic shift following the intervention. Firstly, the consistency between scores on the two depression scales (BDI and HAMD) significantly increased following the intervention, with the correlation coefficient rising from a significant positive correlation before intervention (r_s_= 0.536, P = 0.027) to a strong positive correlation afterwards (r_s_= 0.742, P < 0.001). Secondly, intra-system coordination within HRV emerged post-intervention, SDNN and RMSSD shifted from no significant correlation (r_s_= 0.246, P = 0.342) to a significant positive correlation (r_s_= 0.537, P = 0.026); simultaneously, the negative correlation between LF/HF and HF changed from non-significant (r_s_= -0.296, P = 0.249) to significant (r_s_= -0.667, P = 0.003). Beck Depression Inventory scores exhibited a positive correlation trend with autonomic balance indicators (LF/HF) (r_s_= 0.422, P = 0.091). Concurrently, frontal lobe EEG alpha wave lateralization (FP1-FP2) also showed a positive correlation trend with heart rate (HR) (r_s_= 0.474, P = 0.055). Electroencephalographic indicators maintained high internal consistency both before and after the intervention. Analysis of pre- and post-intervention change values revealed a significant negative correlation between the degree of improvement in individual depressive symptoms (ΔBDI) and the reduction in heart rate (AHR) (r_s_= -0.684, P = 0.002). Concurrently, changes in EEG indices exhibited high levels of synergistic variation (ΔFP1-FP2 and ΔF3-F4, r_s_= 0.922, P < 0.001).

**Table 7:** Comparison of Spearman’s correlation analysis results between variables before and after intervention.

Variable pair	Pre-intervention	Post-intervention	Change value
	Correlation/P-value p	Correlation/P-value p	Correlation/P-value
Psychological scales			
BDI – HAMD	0.536*/0.027	0.742**/<0.001	0.451/0.069
HRV			
SDNN–RMSSD	0.246/0.342	0.537*/0.026	-0.305/0.233
LF/HF – HF	-0.296/0.249	-0.667**/0.003	-0.801**/<0.001
HF-RMSSD	0.252/0.330	0.428/0.087	-0.232/0.371
EEG indicators			
FP1-FP2–F3-F4	0.917**/<0.001	0.563*/0.019	0.922**/<0.001
FP1+F3–FP2+F4	0.949**/<0.001	0.803**/<0.001	0.809**/0.001
Psycho-physiological			
HAMD – HF	0.484/0490	0.264/0.306	-0.374/0.139
BDI – LF/HF	-0.361/0.154	0.422/0.091	0.207/0.424
FP1-FP2 – HR	-0.267/0.301	0.474/0.055	0.108/0.680
BDI-HR	-0.077/0.770	0.085/0.745	-0.684**/0.002
BDI-SDNN	-0.427/0.088	-0.190/0.465	-0.460/0.063

*p < 0.05, **p <0.01

## 4 Discussion

### 4.1 Feasibility of aerobic exercise in treating depressed college students

The etiology of depression is multifaceted and complex. University freshers confront numerous external factors, including a novel environment, altered educational approaches, and shifting interpersonal dynamics, making them more vulnerable to psychological problems like depression and anxiety (27). Current treatment approaches for depression primarily include pharmacotherapy, cognitive behavioral therapy, exercise therapy, and physical therapy. Medications often carry numerous adverse side effects, leading to widespread patient apprehension and refusal of treatment (28). This study employed an nine-week exercise intervention, circumventing the risks associated with pharmacological treatment. It demonstrated that exercise improves the emotional state of depressed university students, accompanied by positive changes in autonomic nervous system function and prefrontal cerebral electrical activity. As a non-pharmacological intervention, exercise demonstrates significant efficacy in treating psychological disorders. It markedly alleviates negative emotions in depressed individuals, increases endogenous neurotrophic factors, and restores receptor hypofunction caused by prolonged negative affect. This promotes brain functional recovery, reduces stigma associated with the condition, and facilitates restoration of normal social functioning. Furthermore, exercise therapy carries a low risk of side effects and exhibits favorable treatment adherence (29). In this study, only three of the 20 depressed university students in the exercise group were unable to complete the eight-week aerobic intervention. The dropout rate for exercise therapy was lower than that observed in previous clinical studies involving conventional medication (24). This study found that exercise improved depressive symptoms and prefrontal cortex function in university students with depression, balanced the autonomic nervous system, and enhanced coordination among these three factors. As a safe, effective, low-cost behavioral intervention with minimal side effects, exercise avoids the adverse effects and high costs associated with medication. It also circumvents the stigma experienced by patients, enhances treatment adherence, and improves brain function. Consequently, exercise therapy is more readily promoted and implemented among university students with mild to moderate depression.

Although this study did not include a control group receiving medication or psychotherapy, the improvement observed in the exercise group on the depression scale (a reduction of approximately 53% in BDI scores) is comparable to the effects reported in some short-term trials of antidepressant medications. Furthermore, the exercise intervention demonstrated unique advantages in improving HRV and frontal lobe alpha lateralization, suggesting it may exert effects through multiple neurophysiological pathways rather than merely alleviating symptoms. Future research could further explore the synergistic effects of exercise in combination with medication and psychotherapy.

### 4.2 Aerobic exercise improves the function of the prefrontal lobes of the brain

Frontal lobe electroencephalogram frontal α-band asymmetry is a significant biological indicator for predicting depression risk and a primary tool for assessing and diagnosing depression (30). The α-band represents a physiological frequency range crucial for normal brain function, prominently present during rest and relaxed concentration. The frontal lobe plays a vital role in regulating executive functions and emotional responses (4). The symmetry of the α-band in the frontal lobe is closely linked to emotional fluctuations in individuals. Patients with major MDD exhibit abnormal EEG patterns characterized by altered frontal α-band symmetry, accompanied by increased beta wave amplitude and power. Left frontal lobe activity correlates with positive emotions, whereas right frontal lobe activity is associated with negative emotional states (31). The study revealed that frontal α frequency band asymmetry can serve as a predictive indicator for depression severity and treatment efficacy. Improving the asymmetry of the frontal α frequency band has been associated with positive treatment outcomes (32). Physical exercise, as an exogenous stimulus, has the potential to restructure the brain and complement depression treatment efforts (33). Exercise interventions have shown to significantly elevate the overall average power of EEG in individuals with depression, consequently ameliorating depressive symptoms (34). Bahmani et al (35) conducted a three-week aerobic training program, involving 60 minutes of exercise per session, for patients with depression. They observed improvements in sleep quality, depression symptoms, fatigue reduction, and cognitive performance. This study further revealed that the exercise group exhibited a reduction in total frontal alpha-band power and significant improvement in frontal alpha-band lateralization compared to pre-intervention levels. These findings suggest that aerobic exercise can serve as an emotion-evoking activity that mediates the power differences between the brain’s left and right hemispheres, facilitating a shift from negative to positive emotions in college students with depression. Post high-intensity exercise, muscles respond to external mechanical stress by producing “endogenous factors” that influence the oscillatory activity and alpha-band symmetry of the prefrontal cortex, thereby impacting emotional changes (34). Regular long-term exercise significantly remodels the cerebral cortex, affecting functional brain network and enhancing autonomic brain function. This adaptation prepares the brain to respond more effectively to exercise-induced stimuli, thereby improving exercise performance (36). In addition, the altered frontal alpha band lateralization in depression may stem from prolonged negative mood, leading to functional impairment in the frontal limbic and subcortical regions (37). Exercise can activate damaged neurons, stimulate thalamo-cortical feedback, and promote the differentiation and growth of serotonergic and dopaminergic neurons (38). This process induces thalamo-cortical circuits, enhancing frontal electroencephalographic activity, thereby alleviating depressive symptoms and aiding the recovery of functional brain networks (39).

### 4.3 Aerobic exercise increases HRV in depressed college students

Depressive syndromes frequently coincide with autonomic nervous system dysfunction. HRV captures the autonomic nervous system’s balance and tension through subtle cardiac cycle differences (40). Research indicates that depression is associated with increased sympathetic and decreased parasympathetic cardiac activity, resulting in reduced HRV, which impairs autonomic regulation and worsens depressive symptoms (41, 42). Additionally, HRV is proposed as a biomarker for assessing treatment efficacy and forecasting depression progression. The standard deviation of normal-to-normal intervals (SDNN) indicates HRV dispersion, while the root mean square of successive differences (RMSSD) measures parasympathetic influence on heart rate. High frequency (HF) correlates with parasympathetic activity, and low frequency (LF) correlates with sympathetic activity. The LF/HF ratio represents the balance between sympathetic and parasympathetic tensions (43). This study found through that an 8-week aerobic exercise intervention led to notable changes in HRV indicators. In the exercise group, heart rate (HR) and the root mean square of successive differences (RMSSD) both increased significantly post-intervention. Conversely, the control group experienced a significant decrease in RMSSD. Regarding frequency-domain indicators, the exercise group showed a significant decrease in normalized low-frequency power (LFn) and a significant increase in normalized high-frequency power (HFn), while the control group exhibited a significant decrease in HFn. These findings suggest that aerobic exercise enhances HRV, improves autonomic nervous function in individuals with depression, and may exert an antidepressant effect. Regular, long-term exercise can elevate HRV in healthy individuals, enhance autonomic nervous system integration, and correct imbalances between the sympathetic and parasympathetic systems, thereby improving autonomic adaptability to external stimuli (44) (45). High-intensity interval training outperforms moderate-intensity aerobic exercise in boosting HRV among depression patients. This form of training enhances cardiac autonomic integration and parasympathetic regulation, mitigating autonomic dysfunction associated with MDD (46). Additionally, antidepressants have been shown to increase HRV in depression patients, thereby modulating autonomic function and alleviating depressive symptoms (47) Combining antidepressants with exercise further augments HRV more effectively than medication alone (48). Dysfunction of the hypothalamic-pituitary-adrenal (HPA) axis and excessive activation of the renin-angiotensin-aldosterone system (RAAS) are key pathogenic mechanisms in depression (49). In depressed patients, an overactive HPA axis intensifies RAAS activity, leading to excessive aldosterone production, heightened sympathetic nervous system activity, and disrupted autonomic balance, resulting in decreased heart rate variability (HRV) (50). Exercise enhances myocardial function, improves parasympathetic regulation of the heart, and mitigates HPA axis overactivity by reducing corticotropin-releasing hormone levels. This, in turn, suppresses RAAS response, decreases aldosterone release, and increases HRV (51). Research indicates a link between the descending projection fibers of the lateral hypothalamic fasciculus and the dorsal motor nucleus of the vagus nerve, involved in parasympathetic regulation. The lateral hypothalamic fasciculus is integral to human emotions and moods (52). Exercise enhances cardiac function, influencing parasympathetic tone and restoring sympathetic-parasympathetic balance. This enhances lateral hypothalamic fasciculus activity, aiding correction of autonomic dysfunction in depression patients.

### 4.4 Comparison of the exercise program in this study with other exercise interventions for depression

The exercise program employed in this study differs in form and design philosophy from most existing depression intervention research. Previous studies predominantly utilized fixed-intensity moderate-to-vigorous continuous aerobic training (3), whereas this study implemented a 9-week aerobic exercise program featuring progressive load. In terms of intervention structure, fixed-intensity programs may compromise participant adherence and increase dropout risk due to inappropriate intensity settings. In contrast, this study employs a progressive load-increase model by gradually increasing exercise sets, elevating target heart rate, and shortening rest intervals. This design aims to provide depressed university students with sustained physiological and psychological adaptation space: the initial low-intensity phase lowers participation barriers and reduces anxiety, while progressively heightened loads may enhance motivation and persistence through continuous achievement and bodily feedback.

Previous research indicates that HIIT, due to its alternating intensity and rest intervals, can effectively stimulate rapid regulation and recovery capacity within the autonomic nervous system (53). Although this study did not directly employ HIIT, its progressive load design similarly seeks to gradually intensify autonomic nervous system stimulation within safe parameters. Compared to fixed-intensity protocols, this dynamic load adjustment may exert unique effects on regulating the heart-brain interaction pathway, thereby potentially improving depression-related physiological and psychological indicators more effectively. However, due to limitations such as the small sample size, this hypothesis requires further validation in future large-scale studies.

## 5 Conclusion

This study investigated the effects of exercise on emotional changes, autonomic nervous system function, and frontal lobe alpha band lateralization in university students with depression. Results indicated that, compared to the control group, the exercise group exhibited significantly reduced depression scale scores, accompanied by enhanced autonomic nervous system function and altered frontal lobe alpha wave lateralization. These findings suggest that regular long-term aerobic exercise may serve as one means of alleviating symptoms in university students with mild to moderate depression. Furthermore, such offline group activity formats could potentially complement existing mental health promotion systems for university students. However, this study is constrained by a small sample size, a short intervention period, and the absence of long-term follow-up. Consequently, the external validity and long-term stability of these findings require further validation. Moreover, traditional offline group exercise interventions face challenges in long-term adherence and program expansion, constituting a significant limitation to their effectiveness as a treatment for depression.

Building upon these findings and limitations, future research should explore two avenues: enhancing the scalability and personalization of exercise programs to validate long-term effects through larger-scale, extended interventions. Concurrently, novel approaches integrating exercise interventions with digital sports platforms could overcome traditional limitations, improve adherence and engagement, and offer university students experiencing depression more personalized and appealing mental health promotion strategies.

## Data Availability

The original contributions presented in the study are included in the article/supplementary material. Further inquiries can be directed to the corresponding author.
